# Neutrophil Extracellular Traps Upregulate p21 and Suppress Cell Cycle Progression to Impair Endothelial Regeneration after Inflammatory Lung Injury

**DOI:** 10.3390/jcm13051204

**Published:** 2024-02-20

**Authors:** Shuainan Zhu, Ying Yu, Qianya Hong, Chenning Li, Hao Zhang, Kefang Guo

**Affiliations:** 1 Department of Anesthesiology, Zhongshan Hospital, Fudan University, Shanghai 200032, China; zzzhushn@163.com (S.Z.); yu.ying1@zs-hospital.sh.cn (Y.Y.); hongqianya2021@163.com (Q.H.); cnli23@m.fudan.edu.cn (C.L.); 2 Shanghai Key Laboratory of Perioperative Stress and Protection, Shanghai 210000, China

**Keywords:** sepsis-induced lung injury, neutrophil extracellular traps, endothelial regeneration, cell cycle

## Abstract

**Background:** Sepsis is a major cause of ICU admissions, with high mortality and morbidity. The lungs are particularly vulnerable to infection and injury, and restoration of vascular endothelial homeostasis after injury is a crucial determinant of outcome. Neutrophil extracellular trap (NET) release strongly correlates with the severity of lung tissue damage. However, little is known about whether NETs affect endothelial cell (EC) regeneration and repair. **Methods**: Eight- to ten-week-old male C57BL/6 mice were injected intraperitoneally with a sublethal dose of LPS to induce acute lung inflammatory injury or with PBS as a control. Blood samples and lung tissues were collected to detect NET formation and lung endothelial cell proliferation. Human umbilical vein endothelial cells (HUVECs) were used to determine the role of NETs in cell cycle progression in vitro. **Results**: Increased NET formation and impaired endothelial cell proliferation were observed in mice with inflammatory lung injury following septic endotoxemia. Degradation of NETs with DNase I attenuated lung inflammation and facilitated endothelial regeneration. Mechanistically, NETs induced p21 upregulation and cell cycle stasis to impair endothelial repair. **Conclusions**: Our findings suggest that NET formation impairs endothelial regeneration and vascular repair through the induction of p21 and cell cycle arrest during inflammatory lung injury.

## 1. Introduction

Sepsis is an extreme host response to infection that causes life-threatening organ damage, with an annual incidence of more than 48 million cases worldwide, and is still a burden to healthcare systems [1]. One of the hallmarks of acute lung injury (ALI) pathogenesis is the disruption of lung endothelial barrier integrity, resulting in widespread endothelial cell (EC) damage, interruption of endothelial adherent junctions, and even death [2,3]. The blood vessels in the lung endothelium lining constitute almost 50% of all lung cells. They are inescapably exposed to invading pathogens and infiltrating leukocytes when they pass through to reach the alveolar parenchyma [4]. Endothelial repair, secondary to the regeneration of ECs, is of great importance for the resolution of inflammation and tissue restoration after severe acute lung injury. When insufficient, a defective endothelium leads to uncontrolled leukocyte infiltration and inflammation, sustained leakage of vital proteins, and ultimately high mortality. Thus, an improved molecular understanding of the mechanisms underlying vascular regrowth in injured lung tissues is required.

Circulating neutrophils are among the first cells to be recruited to defend against invading pathogens, and these cells function through phagocytosis, degranulation, and the release of neutrophil extracellular traps (NETs) [5,6]. NETs are web-like DNA scaffolds inlaid with abundant granular proteins, histones, and other cytosolic components [7]. In the fight against bacterial pathogens, NETs act like a double-edged sword, with NET formation being important for clearing infection but excessive NETs being deleterious to the efficient regeneration of the endothelium and epithelium [8,9,10]. Surviving ECs undertake the necessary proliferative repair. However, according to our previous research, excessive NET formation also impairs tissue repair, probably by affecting wound structure, cellular regeneration and repair, and angiogenesis [11]. Endothelial cells cultured in the presence of NETs from Behçet’s disease patients exhibited a marked decrease in proliferation and an increase in apoptosis [12]. In addition, NETs exert detrimental effects on endothelial migration and tube formation [13]. Overproduction of NETs contributes to poor vascularization and remodeling after stroke recovery [14]. Thus, we wonder whether NETs are involved in both “fighting” and “fixing” in septic injury and recovery as well. However, whether NETs are involved in endothelial proliferation and restoration during sepsis is unknown.

In the present study, we investigated the effects of NETs on endothelial regeneration and vascular repair in a mouse model of LPS-induced acute severe inflammatory lung injury. We hypothesized that targeting NETs using DNase I could protect mice from defective proliferation for vascular repair. To further uncover the mechanisms underlying the NET-induced delay in EC proliferation, we treated human umbilical vein endothelial cells (HUVECs) with NET-containing medium and performed RNA-seq analysis. Importantly, we found that NETs induce p21 upregulation and cell cycle stasis to impair endothelial restoration. Overall, our findings provide a new insight, namely that NETs may participate in both inflammation aggravation and subsequent tissue regeneration, and that NET-mediated cell cycle stasis results in defective endothelial repair.

## 2. Materials and Methods

### 2.1. Ethics Statement and Patients

Blood samples were obtained from healthy donors according to the protocol approved by the Ethics Committee of Zhongshan Hospital, Fudan University. Informed consent was obtained from the patients or their relatives.

### 2.2. Animals

Eight- to ten-week-old male C57BL/6 mice were purchased from Shanghai Laboratory Animal Research Center (Shanghai, China). The mice were housed in a pathogen-free environment in the laboratory animal center of Fudan University. The experiments were conducted in accordance with the guidelines for the care and use of laboratory animals and were approved by the Animal Care Committee of Fudan University.

### 2.3. Endotoxemia Induction

To induce endotoxemia, a model that mirrors the lung injury observed in polymicrobial sepsis, mice were challenged with sublethal LPS (#L2630, Sigma-Aldrich, St. Louis, MO, USA) intraperitoneally at 10 mg/kg and sacrificed at 24 and 72 h for blood and tissue harvesting [15]. To degrade NETs, mice were treated with an intraperitoneal injection of 100 μL of DNase I (1 mg/mL dissolved in saline, 143582, Roche, Basel, Switzerland) starting 1 h after LPS administration and the process was repeated daily until the termination of the experiments [16,17,18]. Mice were randomly assigned to treatment groups with approximately equivalent numbers of mice in each group.

### 2.4. Isolation of Neutrophils and NET Production

Human neutrophils were obtained and purified using the human neutrophil isolation kit (#LZS11131, TBD Sciences, Tianjin, China) as previously described [10]. In brief, blood samples were layered over the neutrophil separation medium and centrifuged at 3000 rpm for 30 min. The lower layer containing neutrophils was aspirated, washed, eliminated erythrocytes, and resuspended in DMEM containing 10% FBS. After counting, neutrophils were seeded in 6-well plates at a density of 1 × 10^6^ cells per mL and stimulated with 100 nM PMA (#MZ2401, MKbio, Shanghai, China) for 4 h. Then, the supernatant was discarded, and the NETs adhered to the bottom were washed, centrifuged, and collected for further use.

### 2.5. Quantification of dsDNA and MPO-DNA Complexes

Ds DNA in the plasma of mice was detected using Quant-iT^TM^ PicoGreen ^®^ dsDNA Reagent and Kits (Invitrogen, Carlsbad, CA, USA). As described, the obtained plasma and diluted standard DNA were added to a Picogreen working solution and incubated for 2 to 5 min at room temperature, protected from light. Fluorescence intensity was measured by a fluorescence microplate reader at a 480 nm excitation wavelength and 530 nm emission wavelength. The amount of DNA was determined from the standard curve.

The concentration of MPO-DNA complexes in the plasma of mice was determined using a capture ELISA kit, as previously reported. An anti-MPO antibody (22225-1-AP, Proteintech, Wuhan, China) was coated overnight at 4 °C for the antibody capture. After blocking with 1% BSA, the plasma was added to the wells with peroxidase-labelled anti-DNA antibody and incubated for 2 h at room temperature. Next, the plate was washed, and peroxidase substrate was added for 40 min of incubation at 37 °C, protected from light. Optical density was determined at 405 nm.

### 2.6. Histopathological Analysis

Lung tissues were inflated via trachea cannula by gentle infusion of fixative 4% paraformaldehyde and placed in fixatives for 24 h and then embedded in paraffin, cut into 4 μm sections, and placed on glass slides. After being dewaxed and rehydrated using dimethylbenzene and an ethanol solution, the sections were subjected to hematoxylin and eosin (H&E) staining to observe the severity of lung injury. A semiquantitative scoring system was used for histological examination [10], which was based on the following criteria: alveolar edema, hemorrhage, leukocyte infiltration, and the thickness of the alveolar wall. From 0 to 3 (0 = normal; 1 = mild; 2 = moderate; 3 = severe), the individual scores of each item were summed to represent the degree of lung injury.

### 2.7. Immunohistochemistry

Lung tissue sections were dewaxed and steamed in the citric acid buffer to expose antigen-binding sites. Subsequently, the sections were blocked with 1% BSA (BD Biosciences, San Jose, CA, USA) and incubated with primary antibodies against Ki67 (1:200, Servicebio, Wuhan, China) overnight at 4 °C. After washing, secondary antibodies were used, followed by diaminobenzidine staining and counterstaining with hematoxylin. The expression of Ki67 was imaged under light microscopy. The Ki67 index was calculated as the average number of Ki67-positive cells per 100 nuclei, and at least 2000 cells were assessed for the calculations [19].

### 2.8. Evans Blue Albumin (EBA) Tracer Measurement of Pulmonary Transvascular Permeability

EBA was performed to evaluate lung vascular injury and permeability of endothelial barriers. Briefly, 2% Evans blue dye (2 mg/kg, Sangon Biotech, Shanghai, China) was injected into the tail vein of the mice at the end of LPS stimulation and allowed to circulate in the blood vessels for 40 min [20]. Intravascular Evans blue was washed with 10 mL of cold normal saline perfusion through the right ventricle. Then, entire lung tissues were immediately isolated, blotted dry, weighed, and homogenized in 1 mL of PBS, after which the Evans blue dye was extracted by mixing with 2 mL of formamide (Sangon Biotech) and incubating at 60 °C for 18 h. Evans blue content was collected after centrifugation at 5000× *g* for 30 min and determined at 620 nm using a microplate reader. Extravasated Evans blue was presented as micrograms of Evans blue dye per g of lung tissue.

### 2.9. EdU Assay of EC Proliferation

The proliferation of ECs was detected using the Beyo-Click^TM^ EdU Cell Proliferation Kit with Alexa Fluor 488 or 594 (Beyotime Biotechnology, Shanghai, China) according to the manufacturer’s instructions. Briefly, approximately 2 × 10^5^ HUVECs were seeded in 6-well plates and treated with the corresponding agents, after which 10 μM 5-ethynyl-2′-deoxyuridine (EdU) reagent was added to each well at the indicated time and incubated for 2 h at 37 °C. The cells were then washed and fixed with paraformaldehyde for 15 min and permeabilized with 0.3% Triton X-100 for another 15 min. After washing, the cells were incubated with the click reaction reagent for 30 min and stained with Hoechst to indicate the nucleus. Images were obtained under an Olympus microscope (Tokyo, Japan), and results were shown as the proportionate of the EdU-positive cells to Hoechst-positive cells determined using Fiji/ImageJ 1.53v (https://imagej.net/ij/index.html, 10 September 2022) software.

For the in vivo EdU labeling assay, 50 mg/kg EdU (ST067, Beyotime Biotechnology) was intraperitoneally injected at the end of LPS stimulation according to the manufacturer’s instructions. Four hours later, lung tissues were quickly harvested and fixed in 4% paraformaldehyde overnight. The next day, the tissues were embedded in paraffin, cut into 4 μm sections, and placed on glass slides. The slides were dewaxed, rehydrated, subjected to antigen retrieval, and then blocked with 1% BSA for 1 h. To determine whether NET formation affects EC proliferation in the lungs, we used CD31 as an EC marker and performed EdU staining [3,21,22]. Lung tissues were immunostained with anti-CD31 antibodies overnight at 4 °C. After washing, the sections were incubated with the click reaction reagent for 30 min and stained with Alexa Fluor^®^ 594-conjugated goat anti-rabbit IgG (1:200, ab150080, Abcam, Cambridge, MA, USA). Hoechst was used for nuclear staining. Images were obtained under an Olympus microscope. EdU^+^ nuclei were quantified for each section and presented as the average number of EdU^+^ nuclei among every 1000 nuclei.

### 2.10. Intracellular EdU Flow Analysis

Mice were intraperitoneally injected with EdU as described above. Lungs were harvested from the mice at the end of the experiments, and whole-lung single-cell suspensions were prepared as described [15,23]. The lungs were perfused with 10 mL cold normal saline through the right ventricle to remove blood in the vasculature. Then, lung tissues were immediately isolated, collected, cut into pieces, and digested with collagenase A. After digestion, the cell suspensions were filtered through a 40 μm cell strainer and treated with red cell lysis buffer to eliminate erythrocytes. Then, the single-cell suspensions were fixed, permeabilized, and incubated with click reaction reagent for 30 min. For proliferative EC analysis, the cell suspensions were stained with the Alexa Fluor 700-conjugated rat anti-mouse CD45 antibody (BioLegend, San Diego, CA, USA) and PE/Cyanine 7-conjugated rat anti-mouse CD31 antibody (BioLegend) for 45 min at 4 °C. A FACSCanto II flow cytometer (BD Biosciences) was used to analyse the results.

### 2.11. Cell Culture and Treatments

HUVECs were obtained from the American Type Culture Collection (ATCC) and cultured at 37 °C in a 5% CO2 humidified incubator. The base medium for HUVECs consisted of DMEM (#11965092, Gibco, Grand Island, NY, USA) supplemented with 10% FBS and 1% penicillin/streptomycin. To investigate the role of NETs in the regulation of the cell cycle, DNase I (100U, #EN0521, Thermo, Waltham, MA, USA) was used to degrade NETs in vitro experiments [17,24]. The NET-containing medium was pretreated with DNase I for 2 h before usage. Lentivirus-NC-RNAi or lentivirus-CDKN1A-RNAi were purchased from Shanghai GeneChem (MOI = 10), and the sequence of CDKN1A-RNAi was 5′-AAGACCATGTGGACCTGTCAC-3′. After transfection, transfection efficacy was assessed by qPCR and Western blot, and the cells were used for further studies.

### 2.12. Cell Viability Assay

For the examination of cell viability, the assays were conducted as described previously. A Cell-Counting Kit 8 (Dojindo, Kumamoto, Japan) was used to measure cell viability at the indicated times according to the manufacturer’s instructions.

### 2.13. Immunofluorescence

HUVECs were fixed with 4% paraformaldehyde for 15 min, permeabilized with 0.3% Triton X-100, and blocked with 1% BSA for 1 h, and incubated with primary antibodies against p-21 (1:200 dilution in blocking buffer, ab109199, Abcam) overnight at 4 °C. The next day, the cells were rinsed three times and stained with Alexa Fluor^®^ 488-conjugated goat anti-rabbit IgG (1:200, A0423, Beyotime Biotechnology) for 1 h, after which DAPI was used to stain the nuclei. Images were taken under an Olympus microscope (Evident, Tokyo, Japan).

### 2.14. Western Blot

For cellular experiments, total proteins were extracted using RIPA lysis buffer supplemented with protease inhibitor cocktails. Then, the samples were separated by SDS-PAGE and transferred to polyvinylidene difluoride (PVDF) membranes, as described before. Subsequently, the membranes were blocked with 5% nonfat milk in TBST and immunoblotted with anti-cleaved caspase3 (#9661S, 1:1000, Cell Signaling Technology, Danvers, MA, USA), anti-p21 (ab109199, 1:1000, Abcam), anti-CCNB1 (ab181593, 1:1000, Abcam), anti-CDK1 (ab133327, 1:1000, Abcam), anti-RB (ab181616, 1:1000, Abcam), anti-p-RB (#8516S, 1:1000, Cell Signaling Technology) primary antibodies, and GAPDH (AC033, 1:30000, ABclonal, Wuhan, China) overnight at 4 °C. Then, the PVDF membranes were washed and incubated with HRP-conjugated anti-rabbit IgG secondary antibody (#7076, 1:2000, Cell Signaling Technology) or HRP-conjugated anti-mouse IgG secondary antibody (#7074, 1:2000, Cell Signaling Technology). Protein expression level was detected using an enhanced chemiluminescence (ECL) kit and photographed under the automatic chemiluminescence image analyzer (#5200, Tanon, Shanghai, China). Relative quantification was analyzed based on Image J 1.53v software.

### 2.15. Cell Cycle Analysis

For cell cycle analysis, control and NET-treated HUVECs were collected and detected at the end of treatment using a cell cycle kit (Invitrogen). Briefly, harvested cells were washed twice with PBS and fixed with ice-cold 70% ethanol overnight at 4 °C. The next day, the cells were washed and added to RNase A and incubated with propidium iodide (PI) for 30 min in a dark environment. The FACSCanto II flow cytometer (BD Biosciences) was used to analyze the ratio of cells in the distribution of phases G1, S, and G2.

### 2.16. Real-Time Quantitative PCR

Total RNA of cells and lung tissues was extracted using TRIzol reagent (#15596018, Invitrogen), and RNA (1 μg) was reverse-transcribed into cDNA using a PrimeScript RT reagent Kit (#RR820A, Takara, Tokyo, Japan). Quantitative real-time PCR was then performed with the sequence detection system (QuantiStudio 5, Applied Biosystems, Carlsbad, CA, USA) using a TB Green PCR Kit (#RR036A, Takara). The primers used were purchased from TSINGKE Biological Technology, and the sequences of the primers are listed in Table S1. Gene expression was normalized to that of GAPDH.

### 2.17. RNA Sequencing (RNA-Seq)

Total RNA was obtained from control-HUVECs and NET-treated HUVECs for RNA-seq analysis. RNA quality and quantity were evaluated using a NanoDrop (Thermo Fisher Scientific, Waltham, MA, USA), and RNA integrity was determined using agarose gel electrophoresis. Poly(A) RNA was subsequently purified and used to generate cDNA libraries, which were subsequently sequenced using an MGISEQ-T7 instrument (BGI). After removing adaptors, poly-N sequences, and inferior-quality reads, clean reads were aligned using HISAT2 software, and the read numbers mapped to each gene were quantified using FeatureCounts. Differential expression analysis was performed using DESeq2, and genes with a fold change >2 and *p* < 0.05 were defined as differentially expressed genes (DEGs).

### 2.18. Detection of Reactive Oxygen Species (ROS)

DCFH-DA (#S0033, Beyotime Biotechnology) was used to determine intercellular ROS production according to the manufacturer’s instructions. HUVECs were incubated for 20 min with 10 μM DCFH-DA. Then, the cells were washed with serum-free DMEM 3 times, and fluorescence intensity reflecting the amount of ROS was measured at excitation and emission wavelengths of 488 nm and 525 nm, respectively, using a multimode microplate reader (Infinite 200 PRO, Tecan, Mannedorf, Switzerland).

### 2.19. Mitochondrial Membrane Potential (MMP)

To detect the MMP, the cells were incubated with 1× tetramethylrhodamine (TMRE) working solution (#C2001, Beyotime Biotechnology) and Hoechst for 15 min at 37 °C. The reagent was discarded and the cells were washed with serum-free DMEM 3 times and imaged under an Olympus microscope.

### 2.20. Statistical Analysis

All experimental results are expressed as the mean ± standard deviation (SD) as indicated in the figure legends. Student’s *t*-tests or one-way analyses of variance (ANOVA) followed by Tukey’s corrections were performed for comparisons between groups. All data were analyzed using GraphPad Prism 9.0, Excel 2016, and ImageJ 1.53v software. A value of *p* < 0.05 was considered to indicate statistical significance. The data are representative of at least three biological replicates. The notation *n* refers to the number of data points used for statistical analyses and data presentation.

## 3. Results

### 3.1. Increased NET Formation of Inflammatory Lung Injury Following Septic Endotoxemia

**Figure 1:**
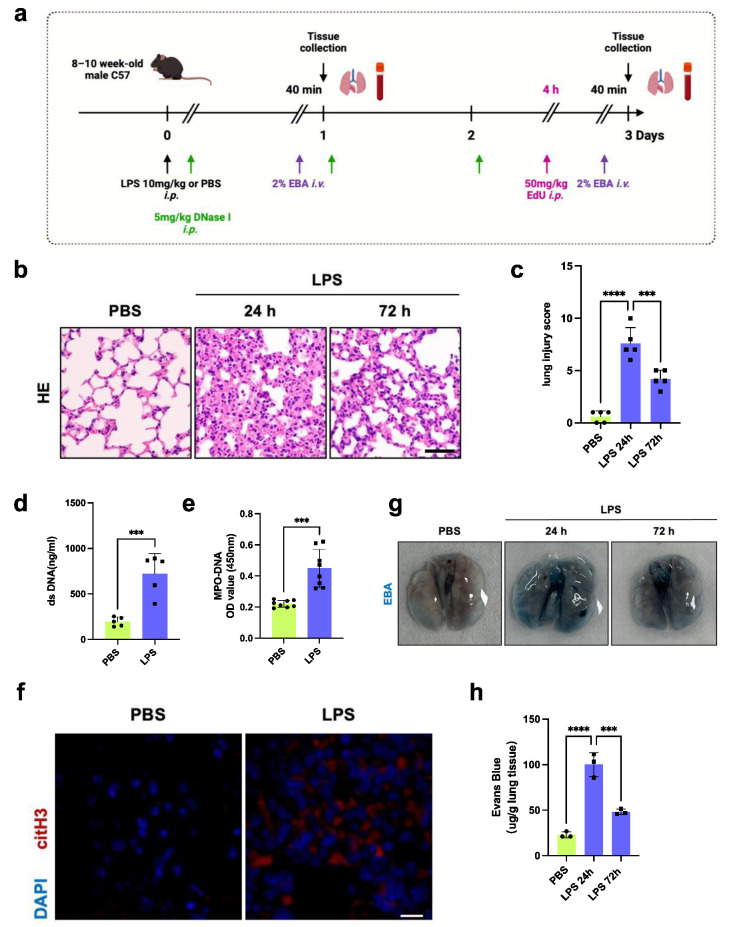
Increased NET formation during inflammatory lung injury following endotoxemia. (**a**) An in vivo experimental paradigm. Mice were challenged with 10 mg/kg LPS or an equal volume of PBS, as indicated by black arrows, and sacrificed at 24 or 72 h after LPS administration. Fifty microliters of 2% EBA was injected into the tail vein 40 min before sacrifice, as shown by the purple arrows. One hundred microliters of EdU was intraperitoneally injected into the mice 4 h before sacrifice, as indicated by the pink arrow. One hundred microliters of DNase I was intraperitoneally injected into the mice daily until the termination of the experiments, as indicated by the green arrow. (**b**) Representative micrographs of H&E staining (*n* = 5; scale bar: 100 μm). (**c**) Semiquantification of lung injury according to H&E staining (*n* = 5). Dots are symbols of the PBS group, and squares are symbols of the LPS group. Same for figures in vivo experiments. (**d**) DsDNA levels in the plasma of mice at 24 h after LPS challenge (*n* = 5). (**e**) Plasma MPO-DNA levels in mice at 24 h after LPS challenge (*n* = 8). (**f**) Representative immunofluorescence images of NETs in lung tissues. CitH3 (red) was used as a marker of NET formation, and the nuclei were stained with DAPI (blue). Scale bar: 20 μm. (**g**) Representative images of the lung 40 min after Evans blue dye injection. (**h**) Lung transvascular permeability was assessed using micrograms of Evans blue dye per gram of lung tissue at the indicated times (*n* = 3). Each bar represents the mean ± SD. Comparisons between two groups were made using unpaired *t* tests (**d**,**e**). Statistical analysis of three or more groups was carried out using 1-way ANOVA (**c**,**g**). *** *p* < 0.001, **** *p* < 0.0001.

We established a model of severe endotoxemia using a sublethal dose of lipopolysaccharide (LPS) intraperitoneal injection, which induced severe inflammatory injury and acute loss of endothelial cells (Figure 1a). After the LPS challenge, mouse lung tissues exhibited severe hemorrhage, edema, thickened alveolar walls, and a large amount of leukocyte infiltration, which was followed by a period of gradual recovery over days (Figure 1b,c). In accordance with our previous studies, neutrophils and NETs played a considerable role in the progression of septic lung injury. We also found that endotoxemic mice exhibited increased levels of dsDNA and MPO-DNA complexes in plasma and citH3 expression in lung tissues, which indicated increased circulating and lung tissue NET formation [10,17,25] (Figure 1d–f). EBA was injected via the tail vein 40 min before the mice were sacrificed to observe the change in pulmonary transvascular permeability after LPS administration. Barrier permeability was obviously impaired at 24 h and gradually restored over 72 h (Figure 1g,h). Collectively, these data showed that increased NET formation and tissue repair occurred simultaneously.

### 3.2. Degradation of NETs Attenuates Lung Inflammation and Facilitates Endothelial Regeneration

**Figure 2:**
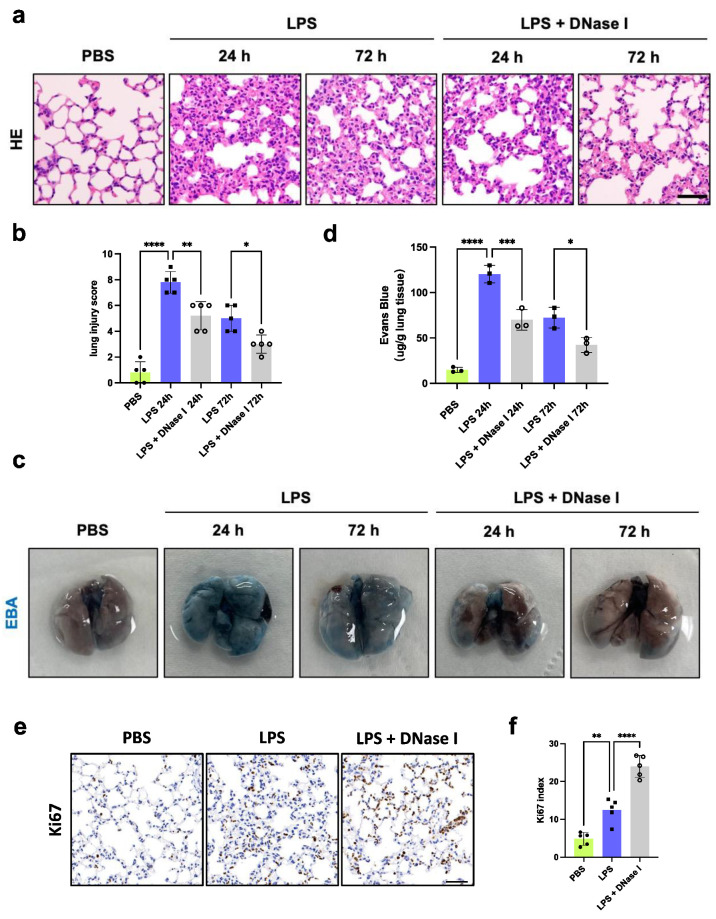

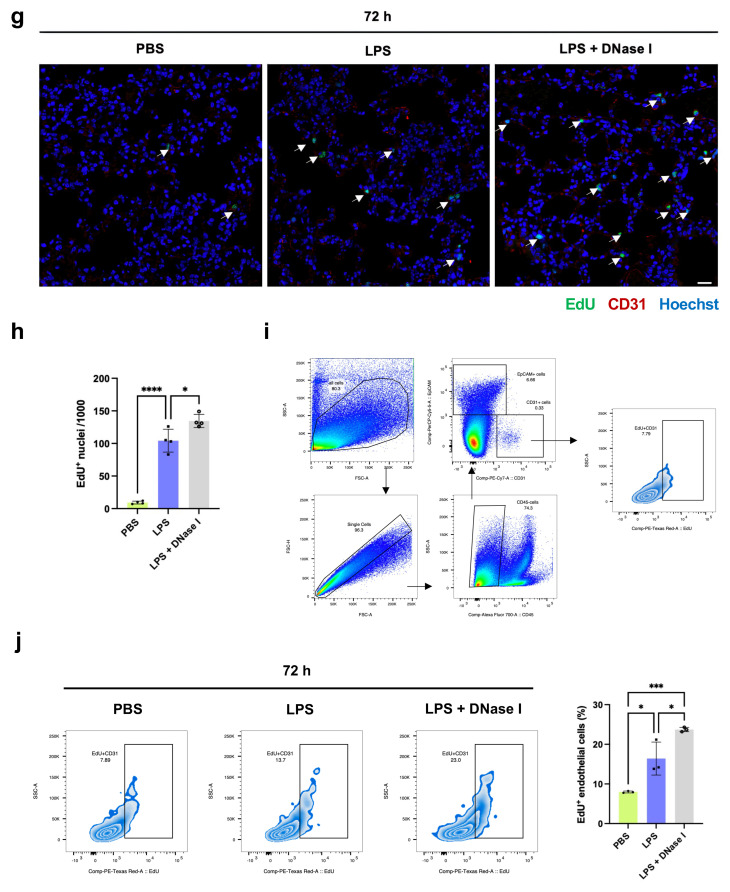
Degradation of NETs attenuates lung inflammation and facilitates endothelial regeneration. (**a**) H&E staining of lung tissues at different time points. Scale bar: 100 μm. (**b**) Semiquantification of lung injury according to H&E staining (*n* = 5). Dots are symbols of the PBS group, and squares are symbols of the LPS group, and circles are symbols of the LPS + DNase I group. (**c**) Representative images of lungs 40 min after Evans blue dye injection. (**d**) Pulmonary transvascular EBA assay demonstrated defective vascular repair in septic mouse lungs, which was rescued by NETs degradation treatments (*n* = 3). (**e**) Representative images of immunohistochemical staining for Ki67 in lung tissues at 72 h after LPS challenge. Scale bar: 50 μm. (**f**) Proportions of Ki67-positive cells in lung tissues were calculated by Fiji/ImageJ software (*n* = 5) (**g**) Representative images of proliferating lung ECs after treatment with PBS, LPS, or LPS and DNase I for 72 h. Green, EdU; red, anti–CD31; blue, Hoechst. Arrows indicate proliferating ECs. Scale bar: 20 μm. (**h**) Graphical representation of the average number of EdU-positive nuclei in the PBS group, LPS group, and DNase I treatment group (*n* = 4). (**i**) Representative gating strategy for flow cytometry on proliferating ECs, which was defined as CD45−EpCAM−CD31+EdU+. (**j**) Representative flow cytometry plots for proliferative ECs in each group and the quantification of proliferative ECs as a percentage of total lung ECs at 72 h after PBS or LPS administration (*n* = 3). Each bar represents the mean ± SD. Statistical analysis of three or more groups was carried out using 1-way ANOVA (**b**,**d**,**f**,**h**,**j**). * *p* < 0.05, ** *p* < 0.01, *** *p* < 0.001, **** *p* < 0.0001.

Next, to determine whether NETs mediate lung inflammation and endothelial regeneration during septic lung injury, we degraded NETs using DNase I i.p. after LPS injection and monitored lung injury and proliferation from Day 1 to Day 3 (Figure 1a). Histopathological analysis of the lung tissues revealed mitigated edema and hemorrhage and decreased leukocyte infiltration after treatment with DNase I at 24 and 72 h after LPS challenge (Figure 2a,b). We also observed the restoration of lung transvascular integrity by measuring EBA flux. As shown in Figure 2c,d, lung barrier integrity was restored more after NET degradation compared to the LPS group at 24 and 72 h. Immunohistochemical staining of Ki67 in lung tissues was much higher in the LPS + DNase I group than in the LPS group, indicating increased tissue regeneration and repair with fewer NETs (Figure 2e,f). To determine lung endothelial repair and proliferation, we performed EdU labeling in vivo [3,21,22,23]. At 72 h after LPS challenge, the proliferation of endothelial cells was greater than that in the PBS control group, demonstrating that the cells underwent initial regeneration after inflammatory injury. There was a significant increase in endothelial proliferation in the lungs after DNase I treatment compared to the LPS group (Figure 2g,h). Meanwhile, we performed intracellular EdU flow analysis to quantify ECs that proliferated at 72 h after PBS or LPS administration, which was defined as CD45−EpCAM−CD31+EdU+ [15,23] (Figure 2i). ECs exhibited a greater proportion of EdU incorporation in the LPS group compared to the PBS control group, which was consistent with the results from immunofluorescence. Mice receiving DNase I treatments after LPS injection showed approximately 25% EdU-positive ECs, but only 14% proliferative ECs in the LPS group, indicating that NETs might impede the proliferation of EC after inflammatory injury (Figure 2j). In summary, these findings suggested that NETs were key components affecting septic lung inflammatory injury and endothelial regeneration.

### 3.3. NETs Impair Endothelial Proliferation by Inducing Endothelial Cell Cycle Arrest

**Figure 3:**
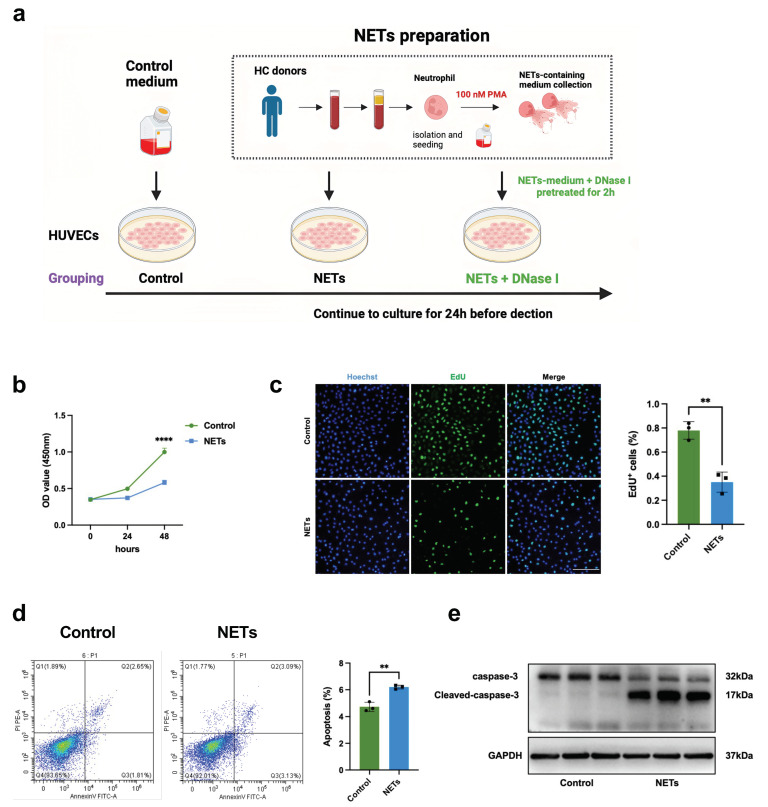

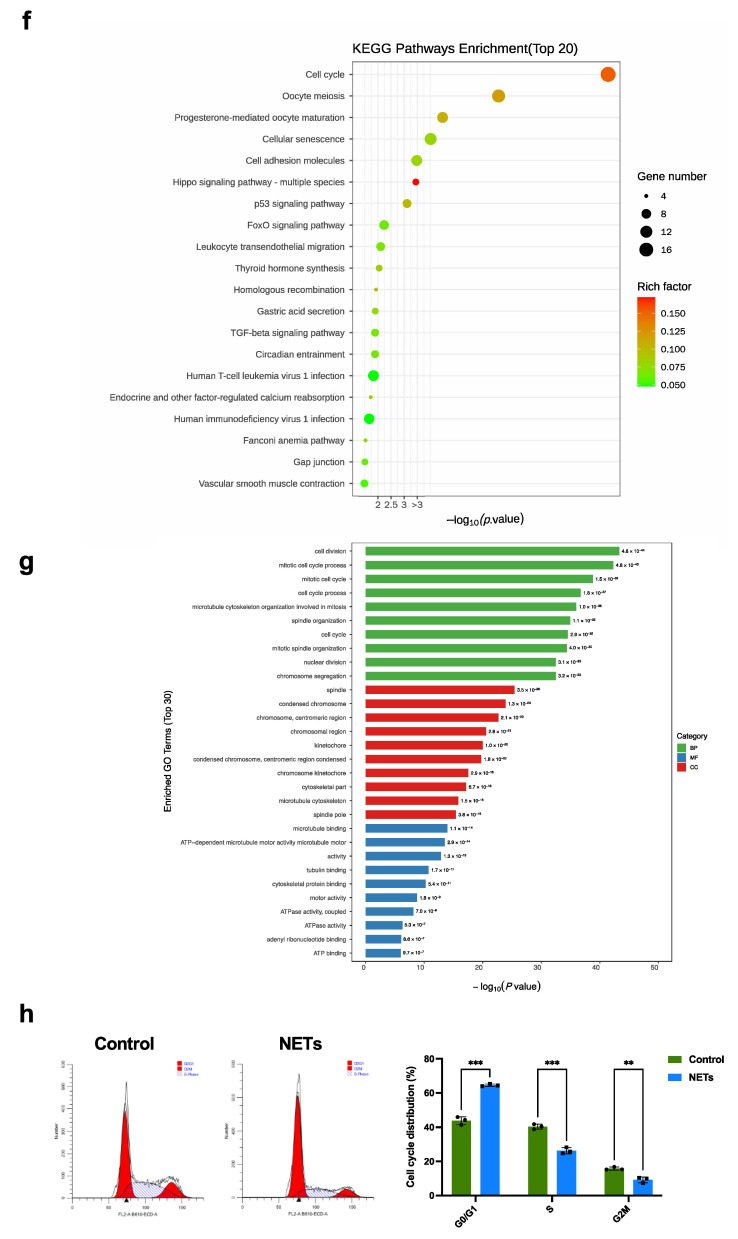
NETs impair endothelial proliferation by inducing endothelial cell cycle arrest. (**a**) A schematic showing the methods used for in vitro experiments. The NET-containing medium was collected and used to stimulate HUVECs. (**b**)The viability of HUVECs in the control and NET-treated groups (*n* = 4). Dots are symbols of the control group, and squares are symbols of the NET-treated group. Same for figures in vitro experiments. (**c**) Representative fluorescence images of EdU stained HUVECs in the control and NET-treated groups. Scale bar: 200 μm. EdU-positive cells (Alexa Fluor 488 azide-labelled; green) and Hoechst-stained nuclei (blue) were obtained. The statistical data were calculated using the EdU-positive cells ratio (*n* = 3). (**d**) Flow cytometry was performed to detect cell apoptosis (*n* = 3). (**e**) Immunoblotting images of cleaved caspase-3 expression in HUVECs (*n* = 3). (**f**) KEGG analysis identified pathways whose expression was altered in control and NET-stimulated HUVECs (*n* = 5). (**g**) GO analysis of DEGs. (**h**) Flow cytometric analysis of the cell cycle in the control and NET-treated groups (*n* = 3). Each bar represents the mean ± SD. Comparisons between two groups were made using unpaired *t* tests (**b**,**c**,**h**). ** *p* < 0.01, *** *p* < 0.001, **** *p* < 0.0001.

We next investigated the critical role of NETs in endothelial cell proliferation in vitro. We collected PMA-stimulated neutrophil medium (PMA is the most commonly used stimulator of NET formation) and treated human umbilical vein endothelial cells (HUVECs) with them (Figure 3a). Compared with those in the control group, the viability of the NET-treated HUVECs was greatly decreased (Figure 3b). We also detected the regeneration capability of HUVECs after NET stimulation by EdU staining. As shown in Figure 3c, the number of EdU-positive cells was significantly lower in the NET-treated group than in the control group, suggesting the involvement of NETs in endothelial regeneration. In addition, HUVECs exposed to NETs exhibited increased apoptosis (Figure 3d,e). We further performed RNA-seq analysis to assess the transcriptional machinery responsible for the NET-triggered impairment of endothelial proliferation. At 24 h after the NETs challenge, Kyoto Encyclopedia of Genes and Genomes (KEGG) analysis and gene ontology (GO) analysis revealed that genes involved in cell cycle progression, cell division, and cellular senescence were markedly enriched in the NET-treated group (Figure 3f,g). These changes were confirmed by cell cycle analysis. We found that NET-treated HUVECs took significantly longer to progress through the S phase, with an accumulation of cells in the G0/G1 phase and a reduced proportion of cells in the S phase and G2M phase (Figure 3h). These experiments revealed the potential role of NETs in endothelial proliferation and regeneration.

### 3.4. NETs Upregulate p21 and Inhibit Cell Cycle-Related Proteins

**Figure 4:**
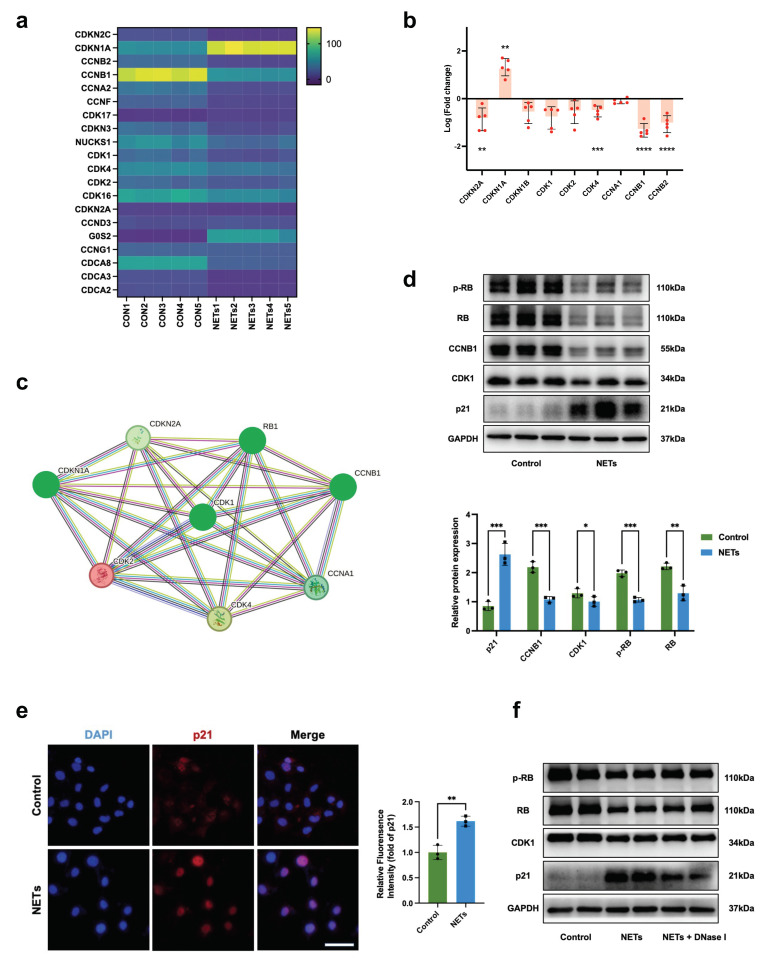
NETs upregulate p21 and inhibit cell cycle-related proteins. (**a**) Heatmap of DEGs implicated in cell cycle modulation after NETs stimulation (*n* = 5). (**b**) Fold change (log) in the expression of cell cycle regulators and differentiation genes in HUVECs treated with NETs normalized to that in control cells (*n* = 5). (**c**) Mev software (4.8.1 version) was used to construct a protein network predicting the interactions between NET-related proteins. (**d**) NETs significantly increased the expression of p21 and decreased the expression of CDK1, RB, p-RB, and CCNB1 (*n* = 3). (**e**) Representative images of immunofluorescence staining of p21 in the control and NET-treated groups. Scale bar: 100 μm. The expression of p21 was assessed by the relative fluorescence intensity (*n* = 3). (**f**) Western blot images of p21, CDK1, RB, and p-RB expression in HUVECs after treatments. Each bar represents the mean ± SD. Comparison between the two groups were made using unpaired *t* tests (**b**,**d**,**e**). ** *p* < 0.01, *** *p* < 0.001, **** *p* < 0.0001.

To confirm the role of NETs in the modulation of the endothelial cell cycle and regeneration, we investigated transcriptome changes implicated in the cell cycle in HUVECs treated with NETs. Several genes implicated in cell proliferation were dysregulated, including CDKN1C, CDKN2A, CDK1, CDK2, CDK4, CCNA1, CCNB1, CCNB2, and CDKN1A, a key modulator of cell cycle progression, was upregulated (Figure 4a). These results were further confirmed by quantitative RT-PCR (Figure 4b). Among these genes, we focused on CDKN1A, which encodes p21 and plays an important role in cell cycle modulation [26]. Several classic downstream targets of p21, including cyclin B1 and the hypophosphorylation form of retinoblastoma (RB), were predicted via STRING software v 12.0 (Figure 4c). Significantly enhanced levels of p21 were observed in NET-treated HUVECs, along with reduced levels of CDK1, CCNB1, RB, and p-RB, suggesting that the p21/CDK1/CCNB1 pathway is dysregulated in HUVECs in response to NETs (Figure 4d). In line with these findings, compared to control HUVECs, NET-treated HUVECs exhibited enhanced immunofluorescence staining for p21 (Figure 4e). Conversely, degradation of NETs using DNase I (1000 U/mL) partially offset this effect (Figure 3a), and we observed reduced p21 expression along with rescued CDK1, RB, and p-RB expression after DNase I treatment (Figure 4f). Collectively, these results indicated that NETs induce cell cycle stasis through upregulation of p21 and the inhibition of cell cycle-related proteins.

### 3.5. CDKN1A Knockdown Protects Endothelial Cells from NET-Induced Regeneration Defects

**Figure 5:**
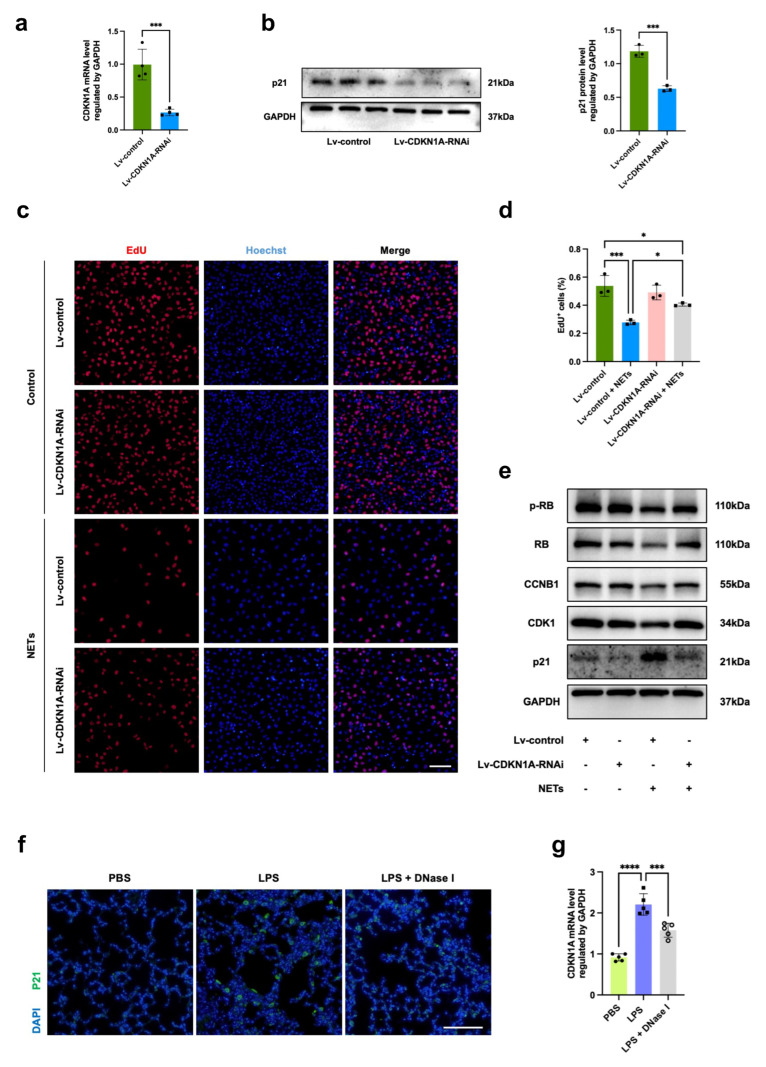
CDKN1A knockdown protects endothelial cells from NET-induced regeneration defects. (**a**) Relative mRNA levels of CDKN1A in HUVECs transfected with Lv-negative control (NC)-RNAi or Lv-CDKN1A-RNAi (*n* = 4). (**b**) Western blot images of p21 expression in HUVECs transfected with Lv-NC-RNAi or Lv-CDKN1A-RNAi (*n* = 3). (**c**) Representative images of EdU-stained HUVECs transfected with Lv-NC-RNAi or Lv-CDKN1A-RNAi and stimulated with control or NETs. Scale bar: 200 μm. EdU-positive cells (Alexa Fluor 594 azide-labelled; red) and Hoechst-stained nuclei (blue) were obtained. (**d**) Quantification of EdU-positive cells in different groups (*n* = 3). (**e**) Western blot images of p21, CDK1, RB, and p-RB expression in HUVECs transfected with Lv-NC-RNAi or Lv-CDKN1A-RNAi and stimulated with control or NETs. (**f**) Representative immunofluorescence micrographs of p21 (green) staining in lung tissues at 24 h after LPS challenge. Nuclei were stained with DAPI (blue). Scale bar: 100 μm. (**g**) CDKN1A mRNA expression in lung tissues at 24 h after LPS injection was detected in the PBS, LPS, and LPS + DNase I groups (*n* = 5). Dots are symbols of the PBS group, and squares are symbols of the LPS group, and circles are symbols of the LPS + DNase I group. Each bar represents the mean ± SD. Comparisons between two groups were made using unpaired *t* tests (**a**,**b**). Statistical analysis of three or more groups was carried out using one-way ANOVA (**d**,**g**). * *p* < 0.05, *** *p* < 0.001, **** *p* < 0.0001.

To further investigate whether p21 upregulation plays a role in NET-mediated cell regeneration stasis, we transfected HUVECs with Lv-CDKN1A-RNAi to knock down p21 expression, and its efficacy was assessed by quantitative RT-PCR and Western blotting (Figure 5a,b). In response to NETs, compared with the negative control treatment, the knockdown of CDKN1A partially rescued impaired endothelial proliferation compared to the negative control group, establishing a critical role for p21 in NET-mediated endothelial repair and regeneration (Figure 5c,d). Consistent with these findings, we detected increased expression of CDK1, CCNB1, RB, and p-RB protein expression in the CDKN1A knockdown group (Figure 5e). Additionally, p21 protein as well as mRNA expression were decreased after DNase I treatment in vivo, revealing that NETs modulate p21 expression (Figure 5f,g). Taken together, these data further support the indispensable role of p21 in NET-mediated defective tissue repair.

### 3.6. NETs Impair Mitochondrial Bioenergetics via p21/Cyclin B1/CDK1

**Figure 6:**
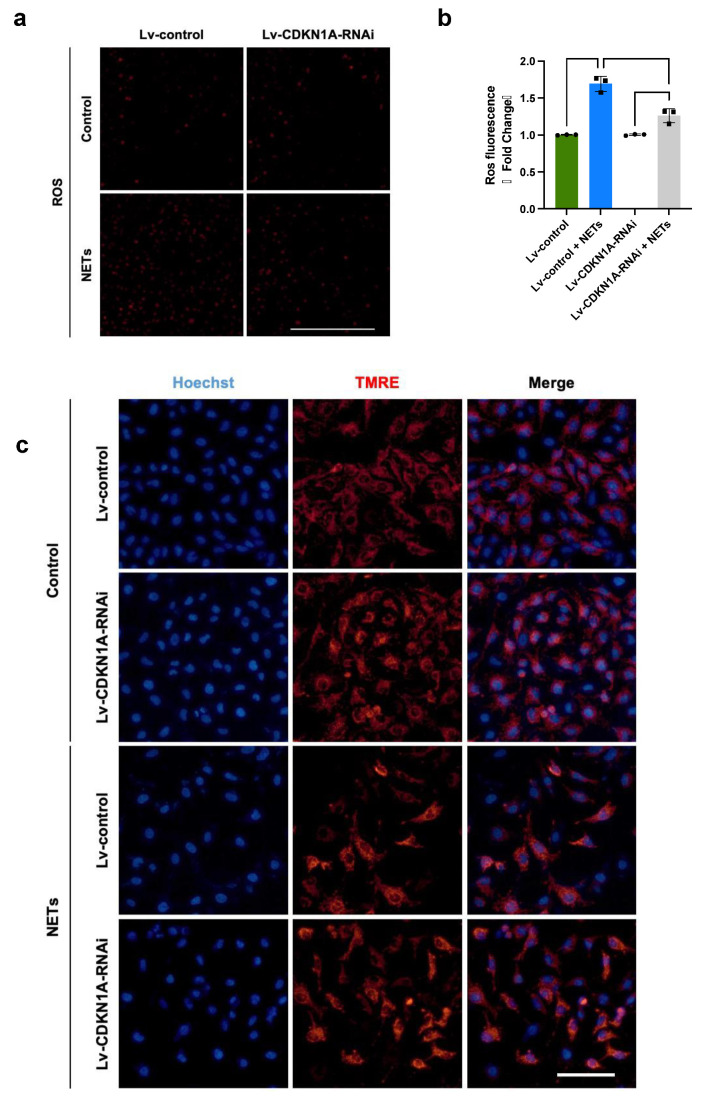
NETs impair mitochondrial bioenergetics via p21/cyclin B1/CDK1. (**a**) Representative images of ROS in control or NET-stimulated HUVECs transfected with Lv-NC-RNAi or Lv-CDKN1A-RNAi. Scale bar: 500 μm. (**b**) Detection of ROS by a multimode microplate reader at excitation and emission wavelengths of 488 nm and 525 nm, respectively (*n* = 3). (**c**) Representative images of TMRE staining of control or NET-stimulated HUVECs transfected with Lv-NC-RNAi or Lv-CDKN1A-RNAi. Scale bar: 100 μm. Statistical analysis of three or more groups was carried out using one-way ANOVA (**b**). ** *p* < 0.01, *** *p* < 0.001, **** *p* < 0.0001.

The mitochondria provide the majority of cellular energy and tightly coordinate the energy consumption for cell cycle progression [27]. Recent findings have revealed that CCNB1/CDK1 complex-mediated mitochondrial bioenergetics synchronize the communication between mitochondrial metabolism and cell cycle progression events [28]. The localization of mitochondrial CCNB1/CDK1 promotes the G2/M transition by enhancing ATP generation and mitochondrial biogenesis [29,30]. We hypothesized that NET-induced p21 interferes with endothelial mitochondrial homeostasis through dysregulation of the CCNB1/CDK1 complex, impairing cell proliferation and repair. Thus, we further investigated mitochondrial homeostasis under the stimulation of NETs. We found that HUVECs treated with NETs showed a 1.6-fold increase in ROS generation, whereas the knockdown of CDKN1A attenuated the overproduction of ROS (Figure 6a,b). Detection of mitochondrial potential dye TMRE showed that the MMP in HUVECs treated with NETs was significantly lower than that in HUVECs not stimulated with NETs (Figure 6c). Importantly, the silencing of CDKN1A prevented the NET-induced reduction in MMP. Taken together, these findings connoted that NETs disrupt mitochondrial homeostasis probably through modulation of p21/CCNB1/CDK1, thus impairing endothelial regeneration.

## 4. Discussion

EC proliferation and vascular repair are of great importance to resolving lung inflammation and restoring of barrier integrity post-sepsis. Here, we revealed the additional role of NETs in endothelial regeneration and vascular repair after severe inflammatory lung injury. NETs shift multiple pathways involved in cell cycle progression and cell division, thereby suppressing vascular repair in ECs. We showed that NETs augment p21 expression and inhibit cyclin-mediated cell cycle progression and cell proliferation. In contrast, the knockdown of CDKN1A in HUVECs partially reversed NET-induced cell cycle arrest and mitochondrial bioenergetic impairments.

ECs have the capacity to respond to cell loss by compensatory proliferation after severe inflammatory injury [3]. Genetic lineage-tracing studies have shown that the majority of regenerative ECs are derived from tissue-resident ECs rather than from circulating ECs [31]. Thus, the surviving ECs after injury deserve more attention to avoid long-lasting stimulation and impairment of proliferative capacity. After injury, the surviving cells are initiated to proliferate through the cell cycle. Cell cycle progression maintains cell proliferation and cell survival and is tightly modulated by cell cycle regulators. However, in the occurrence of sepsis or endotoxemia, surviving pulmonary vascular ECs suffer from defective repair and proliferation when inflammatory stimuli persist or when there is a deficiency of the critical intrinsic proliferative regulators [3,21,32,33]. These cells exhibited a stagnant cell cycle and decreased expression of the cell cycle-related proteins, including cyclin E and cyclin D, resulting in impaired pulmonary vascular repair and poor outcomes [15,34]. In addition, based on a public microarray dataset analysis, pathways such as the mitotic cell cycle and alterations in several hub genes of the cell cycle were closely related to the progression of sepsis-related acute respiratory distress syndrome [35]. These studies highlighted that steady cell cycle regulation and progression are essential for endothelial proliferation and regeneration after injury. In accordance with these findings, our work again strengthened the crucial role of cell cycle progression in endothelial repair post-lung injury and revealed the role of “inflaming” NETs in the modulation of cell cycle stasis via upregulation of p21.

P21 is a significant modulator of cell cycle progression and functions in the turnover of mature ECs and neovascularization [36]. It has been found that p21 expression is increased after intraperitoneal sepsis in rats and is correlated with impaired recovery of renal and hepatic function [37,38,39]. We did observe increased p21 in injured lung tissues during LPS challenge, and the expression of p21 was affected by NETs. Consistent with the in vivo experiments, degradation of NETs using DNase I alleviated p21 activation and restored the expression of cyclins. Furthermore, knockdown of CDKN1A partially protected ECs from NET-induced defects in proliferation. Our present study again strengthened the sustained inflammatory stimulation of the NETs, which impairs EC regeneration in a cell cycle-dependent manner. Sustained inflammation after infection can suppress the transcription factors implicated in the developmental angiogenesis of ECs, resulting in a defective proliferation capacity, strongly decreased capillary oxygen saturation, and increased mortality following lung injury [3,23]. NETs are large, extracellular, web-like structures composed of cytosolic and granule proteins, DNA scaffolds, and histones; these components can directly impair cell integrity or interact with cell-intrinsic transcriptional pathways to reprogram cell fate [7,8,9,10]. Our transcriptome data also suggested that NETs change the cell cycle progression-related pathways in ECs, providing additional evidence that inflammation affects intrinsic endothelial proliferation factors. However, further studies are needed to determine the molecular mechanisms and interventions involved in NET-induced revascularization impairment and to profile more specific characteristics of altered ECs.

Furthermore, the enrichment of the cellular senescence pathway in NET-treated HUVECs, combined with the significant upregulation of p21 and stasis of the cell cycle hinted at alterations in the cellular senescence program, as p21 was one of the key regulators of cellular senescence [40,41,42]. However, we did detect increased apoptosis after NET stimulation. This might be inconsistent with resistance to apoptosis, which is another feature of cellular senescence. The majority holds the view that senescent cells activate anti-apoptotic and pro-survival programs targeted at reducing the effects of DNA and protein damage [43,44]. Although apoptosis occurs in response to even greater stress than is required for senescence, senescence-associated secretory phenotype (SASP) factors could initiate apoptosis of surrounding cells [45,46]. Nevertheless, other features of endothelial cell senescence under stimulation of NETs were not explored in the present study. Whether increased apoptosis results from endothelial cell senescence or cytotoxicity of NETs or both needs more research to determine.

In addition, p21 is also a central regulator of innate and adaptive immune responses independent of cell cycle regulation [47]. Moreover, p21 deficiency increases NF-κB activation and pro-inflammatory cytokine production through macrophage reprogramming modulation, thus exacerbating septic shock [48,49]. In addition, p21 favors autophagy via interaction with light chain 3B (LC3B), a key component of autophagosomes [50,51], to improve the outcome of cardiac function post-sepsis [52]. However, additional studies are needed to substantiate the role of p21 in sepsis-induced injury and recovery.

Neutrophils and NETs could persist at an elevated level long after tissue injury and infections, which provides the possibility of NETs affecting tissue remodeling and recovery. In a mouse model of cerebral stroke, researchers found increased neutrophil infiltration that peaked at 3 days and persisted to 14 days in the ischemic cortex, as well NET formation in brain tissues and circulation. Thus, sustained NETs impeded the cerebrovascular remodeling of brain tissues and functional recovery after stroke [14]. In addition, a subset of severe COVID-19 survivors exhibited sustained neutrophil-associated immune signatures including elevated chemokines, proteases, and markers of NETs that were detectable in the plasma and the upper airway for a long period [53,54]. These findings indicate an incomplete resolution of NETs after acute tissue damage or infections. In fact, the role of NETs in tissue repair has been widely studied in wound healing, particularly in diabetes-induced delayed wounds. Anti-NET therapies, including degradation using DNase I and inhibition of NET formation using Peptidyl arginine deiminase 4 (PAD4) inhibitors [55], have been confirmed to be effective at accelerating wound closure and healing [56,57,58]. The inhibitory effects of NETs on EC proliferation and angiogenesis have been studied in several disease models. Our work extended the understanding of NETs in tissue repair to the type of sepsis-induced lung injury, and we found that NETs affect endothelial regeneration and vascular restoration in a cell cycle-dependent manner after lung injury. These findings could lead to additional insights into the role of NETs in tissue repair and provide potential therapeutic targets for defective endothelial proliferation after acute lung injury.

The present work has several shortcomings. Additional research is needed to verify the link between NETs and the cell cycle or cell senescence. Our research focused only on ECs, while the repair and proliferation of lung epithelial cells after acute lung injury, especially type II alveolar epithelial cells, are equally important [59]. Our experiments were performed in only a single cell line; due to the heterogeneity of the endothelial system, validation using isolated mouse lung ECs, human lung microvascular ECs, and genetic tracing systems is needed in the future. Furthermore, given the complex role of p21 in other conditions, specific knockdown of p21 in ECs will provide insight into the role of p21 in cell restoration and regeneration after inflammatory injury. Vascular repair involves two processes, endothelial regeneration and the reestablishment of endothelial junctions. We focused on the former in our current work and provided multiple lines of evidence to illustrate the inhibitory role of NETs in EC proliferation. However, if and how the latter is affected by NETs after injury need more research. Finally, additional preclinical septic models need to be used to verify our findings in view of the recognized heterogeneity of sepsis [60].

In summary, our findings are the first to report that NETs are involved in both “fighting” and “fixing” during sepsis-related acute lung injury. We found that NET formation impaired endothelial regeneration and vascular repair through the induction of p21 and cell cycle stasis. Conversely, degradation of NETs with DNase I or knockdown of CDKN1A in ECs has a protective effect on promoting cell proliferation and restoration.

## Data Availability

All relevant data are available within this published article and Supplementary Materials; further inquiries can be directed to the corresponding author.

## Supplementary Materials

The following supporting information can be downloaded at: https://www.mdpi.com/article/10.3390/jcm13051204/s1, Table S1: Primer sequences for RT-qPCR.
