# Supplementary material for: Neutrophil Extracellular Traps Upregulate p21 and Suppress Cell Cycle Progression to Impair Endothelial Regeneration after Inflammatory Lung Injury
Source: J Clin Med. 2024 Feb 20;13(5):1204. doi: 10.3390/jcm13051204 (PMC10931969; doi:10.3390/jcm13051204)
Supplement: Supplementary file 1 [file jcm-13-01204-s001.zip › jcm-2789728-supplementary.pdf]

Supplementary Table S1. Primer sequences for RT-qPCR.

Gene Name	Forward	Reverse
Human-CDKN1A	TGTCCGTCAGAACCCATGC	AAAGTCGAAGTTCCATCGCTC
Human-CDKN2A	GGGTTTTCGTGGTTCACATCC	CTAGACGCTGGCTCCTCAGTA
Human-CDKN1B	AACGTGCGAGTGTCTAACGG	CCCTCTAGGGGTTTGTGATTCT
Human-CDK1	AAACTACAGGTCAAGTGGTAGCC	TCCTGCATAAGCACATCCTGA
Human-CDK2	CCAGGAGTTACTTCTATGCCTGA	TTCATCCAGGGGAGGTACAAC
Human-CDK4	ATGGCTACCTCTCGATATGAGC	CATTGGGGACTCTCACACTCT
Human-CCNA1	GAGGTCCCGATGCTTGTCAG	GTTAGCAGCCCTAGCACTGTC
Human-CCNB1	AATAAGGCGAAGATCAACATGGC	TTTGTTACCAATGTCCCCAAGAG
Human-CCNB2	CCGACGGTGTCCAGTGATTT	TGTTGTTTTGGTGGGTTGAACT
Human-GAPDH	GGAGCGAGATCCCTCCAAAAT	GGCTGTTGTCATACTTCTCATGG
Mouse-TF	CTGGAAAAACAAGTGCTTCTCG	ACAGAGAGGACCTTTGCTTCA
Mouse-IL-1β	GAAATGCCACCTTTTGACAGTG	TGGATGCTCTCATCAGGACAG
Mouse-IL-6	CTGCAAGAGACTTCCATCCAG	AGTGGTATAGACAGGTCTGTTGG
Mouse-TNF-α	CAGGCGGTGCCTATGTCTC	CGATCACCCCGAAGTTCAGTAG
Mouse-GAPDH	TGACCTCAACTACATGGTCTACA	CTTCCCATTCTCGGCCTTG
